# Unraveling the Complex Polymorphic Crystallization Behavior of the Alternating Copolymer DMDS-*alt*-DVE

**DOI:** 10.1021/acsapm.3c00684

**Published:** 2023-06-09

**Authors:** Valentina Pirela, Justine Elgoyhen, Radmila Tomovska, Jaime Martín, Cuong Minh Quoc Le, Abraham Chemtob, Brahim Bessif, Barbara Heck, Günter Reiter, Alejandro J. Müller

**Affiliations:** † POLYMAT and Department of Polymers and Advanced Materials: Physics, Chemistry, and Technology, Faculty of Chemistry, University of the Basque Country UPV/EHU, Paseo Manuel de Lardizabal 3, Donostia-San Sebastián 20018, Spain; ‡ Institut de Sciences des Matériaux de Mulhouse (IS2M), UMR CNRS 7361, Université de Haute-Alsace, 15 rue Jean Starcky, Mulhouse, Cedex 68057, France; § Institute of Physics, University of Freiburg, Hermann-Herder-Str. 3, Freiburg 79104, Germany; ∥ IKERBASQUE, Basque Foundation for Science, Plaza Euskadi 5, Bilbao 48009, Spain; ⊥ Campus Industrial de Ferrol, CITENI, Esteiro, Universidade da Coruña, Ferrol 15403, Spain

**Keywords:** polymorphism, polythioethers, crystallization, fast scanning chip calorimetry, microcalorimetry

## Abstract

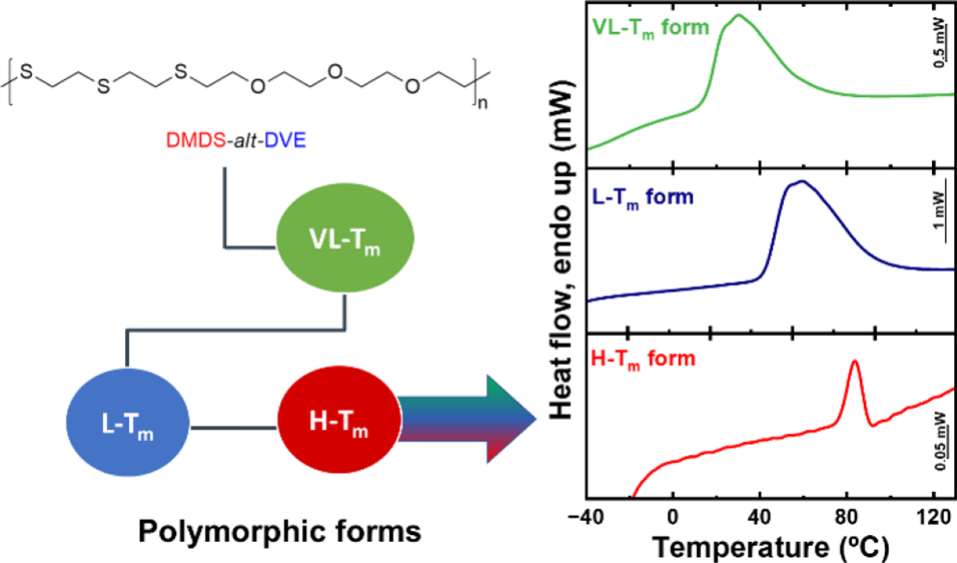

A complex crystallization behavior was observed for the alternating copolymer DMDS-*alt*-DVE synthesized via thiol–ene step-growth polymerization. Understanding the underlying complex crystallization processes of such innovative polythioethers is critical for their application, for example, in polymer coating technologies. These alternating copolymers have polymorphic traits, resulting in different phases that may display distinct crystalline structures. The copolymer DMDS-*alt*-DVE was studied in an earlier work, where only two crystalline phases were reported: a low melting, L – *T*_m_, and high melting, H – *T*_m_ phase. Remarkably, the H – *T*_m_ form was only achieved by the previous formation and melting of the L – *T*_m_ form. We applied calorimetric techniques encompassing seven orders of magnitude in scanning rates to further explore this complex polymorphic behavior. Most importantly, by rapidly quenching the sample to temperatures well below room temperature, we detected an additional polymorphic form (characterized by a very low melting phase, denoted VL – *T*_m_). Moreover, through tailored thermal protocols, we successfully produced samples containing only one, two, or all three polymorphs, providing insights into their interrelationships. Understanding polymorphism, crystallization, and the resulting morphological differences can have significant implications and potential impact on mechanical resistance and barrier properties.

## Introduction

Polymerization technologies have recently focused on thiol–ene chemistry.^1−8^ Historically, this polymerization approach has been used mainly in bulk or solution for applications such as coatings or surface modification.^2,3,5,7^ Aiming to make the process more eco-friendly, there has been a growing interest in thiol–ene polymerization in aqueous dispersed media, such as emulsion and miniemulsion.^4,6,8−11^ Le et al. reported the synthesis of polythioethers with high sulfur content. Such polymers have the ability to crystallize, yielding semicrystalline properties^9^ which are directly related to their chemical and mechanical resistance.^12^ The characterization of newly synthesized materials, mainly when specific applications are targeted such as barrier coatings or materials with high mechanical resistance, requires a comprehensive understanding of their crystallization behavior.

The alternating copolymer DMDS-*alt*-DVE studied in this work is derived by alternatingly linking monomers of di(ethylene glycol) divinyl ether (DVE) and 2,2′-dimercaptodiethyl sulfide (DMDS). This polythioether exhibits different polymorphic forms. In general, polymorphic materials are substances that can form several distinctly different crystalline phases.^13−15^ Polymorphism can profoundly impact the mechanical, thermal, and functional properties of a material,^15−17^ and it is a highly researched topic in fields ranging from polymer science to electronics and biology.^18^ The crystallization behavior and phase transformations of polymorphic materials are influenced by an extensive array of factors, such as temperature variation, additives, solvents, or nucleating agents.^14,17^ A specific crystal form will develop due to an interplay of thermodynamics and kinetics directly related to the processing conditions. For instance, kinetically-controlled solidification may generate metastable structures with high values in free energy, whereas thermodynamically dominated crystallization typically leads to more stable structures.^19^ Typically, the nucleation stage decides which phase will form. However, in some cases, a transformation from a metastable to a more stable form may be observed,^17,20^ a process often causing complications in industrial applications of these materials.

Materials exhibiting polymorphism are sometimes considered problematic as controlling polymorph formation can be difficult.^16,17^ However, in recent years, significant advances in implementing these materials in unprecedented applications have been achieved by exploiting and understanding the behavior of each polymorphic form.^16−19^ The discovery of unknown polymorphic forms, with potentially different crystalline structures, and a profound understanding of how they can be achieved and controlled, allow for widening the spectrum of properties relevant to an array of applications.^21^ For instance, isotactic poly(1-butene) (iPB) is a polymorphic material that can exist in different crystalline forms; when crystallized from the melt, it forms unstable tetragonal form II crystals. However, upon aging at room temperature, iPB will slowly transform into the more stable hexagonal form I crystals. The transformed crystals are reported to have enhanced elastic properties and a higher melting temperature, which makes i-PB highly sought after for various applications, including tubes, water pipes, and pressurized tanks.^22−26^ In addition, isotactic polypropylene (iPP) is known to exhibit a monoclinic α-phase, a trigonal β-phase, and the orthorhombic γ-phase. iPP mainly crystallizes into the more thermodynamically stable α-form when crystallizing from the melt; however, by introducing β-nucleating agents, the β-form is obtained, which is reported to have higher ductility, higher impact resistance, and better weldability than the α-form.^27−30^

In this work, we focus on the tunability of the alternating copolymer DMDS-*alt*-DVE by investigating the calorimetric behavior of its various polymorphs in depth. By employing experiments spanning seven orders of magnitude in scanning rates, we comprehensively understand the structural transitions within the copolymer. We utilize a microcalorimeter (μ-differential scanning calorimeter, μ-DSC) for low scanning rates, a conventional DSC for intermediate rates, and a fast scanning chip calorimeter (FSC) for very fast rates. The morphology is examined using polarized light optical microscopy (PLOM), while the crystalline structure is analyzed through wide-angle X-ray scattering (WAXS). Our primary focus is to comprehensively understand all possible structural transitions of the DMDS-*alt*-DVE alternating copolymer and identify reliable thermal protocols that enable the tuning of properties by accessing different combinations of polymorphic phases. This knowledge is essential for maximizing the potential of this copolymer in various applications.

## Experimental Section

### Materials

**Scheme 1:**
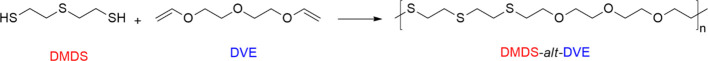
Reaction for the Formation of DMDS-*alt*-DVE

The DMDS-*alt*-DVE alternating copolymer was synthesized via thiol–ene step-growth polymerization between 2,2′ DMDS and a diene di(ethylene glycol) DVE (Scheme 1).

The polymer used in this study has a number average molecular weight (*M*_n_) of 9.8 kDa, a weight average molecular weight (*M*_w_) of 21.9 kDa, and a polydispersity (*Đ*) of 2.23. The synthesis and molecular weight determination are described in the Supporting Information (SI).

### μ-DSC

The measurements were conducted using a Setaram micro-DSC (Micro-Calvet) connected to an Intracooler III under a nitrogen atmosphere with 20 mL/min flow. The samples measured weighted ca. 10 mg. Non-isothermal experiments were conducted where the samples were heated and cooled at a rate of 0.2 °C/min (0.0033 °C/s). Samples were heated from 25 °C to a temperature thirty degrees above the melting point (*T*_m_ + 30 °C) to ensure erasing thermal history for 3 min, then cooled to a temperature below the glass transition temperature (*T*_g_), and subsequently heated thirty degrees above *T*_m_. The relevant temperatures to consider are the crystallization temperature (*T*_c_), the melting point (*T*_m_), and the glass transition temperature (*T*_g_).

### DSC

The experiments were performed using a PerkinElmer DSC 8500 connected to an Intracooler III under a nitrogen atmosphere with 20 mL/min flow. The DSC 8500 was calibrated with indium and tin standards. The samples measured were ca. 5 mg. The Pyris software was used to analyze the data. The samples were heated and cooled at 20 °C/min (0.33 °C/s) during non-isothermal experiments. The sample was heated from room temperature (RT = 25 °C) to a temperature ca. thirty degrees above *T*_m2_ (i.e., 120 °C) for 3 min to completely erase thermal history. The sample was then cooled to a temperature below the glass transition temperature (*T*_g_ ≈ −45 °C) and heated again to *T*_m2_ + 30 °C.

### FSC

FSC experiments were performed on a Mettler-Toledo Flash DSC 2+ device. The equipment was connected to a Huber TC-100 intracooler, permitting scans of up to 40,000 °C/s. The MultiSTAR UFS1 (24 × 24 × 0.6 mm^3^) chip sensors were conditioned and corrected before use according to the Flash DSC 2+ specifications. Measurements were carried out under a nitrogen atmosphere with a constant flow rate of 80 mL/min. It is important to notice that for FSC measurements, the sample mass is in the nanogram scale of magnitude. The STARe software was used to analyze the data. The Results and Discussion section describes the different protocols employed in detail; rates used for this technique ranged from 1 to 10,000 °C/s.

### PLOM

Experiments were performed on a polarized light optical microscope, Olympus BX51 (Olympus, Tokyo, Japan), with an Olympus SC50 digital camera coupled to the microscope. The PLOM was equipped with a Linkam-15 TP-91 hot stage Linkam, Tadworth, U.K., connected to a liquid nitrogen cooling system that was used to observe the morphology of the sample. Films of around 50 μm thickness were prepared by melting the sample between two glass slides.

### WAXS

Experiments were performed using a D500 X-ray powder diffractometer (Siemens, Germany) in reflection mode (θ–2θ scans) with a Cu-Kα radiation source (λ = 1.54 Å) and a scintillation counter at an angular resolution slightly better than 0.1°. The diffractometer was equipped with an evacuated temperature controlled TTK sample chamber (Paar, Austria). To achieve sub-ambient temperature ranges, the chamber was connected to a liquid nitrogen reservoir. The polymer powder, which scattered isotropically, was deposited on an aluminum plate (fabricated in the lab) and placed on a brass block. The temperature was varied by resistive heating through controlling the current. The temperature was measured by a thermometer at the bottom of the heated brass block. This temperature was calibrated to the sample temperature by measuring the actual temperature at the surface of the polymer samples in a control experiment using an external thermocouple (Mini Dual K/J Thermometer, Uni-T, Munich, Germany). Data points of the XRD patterns obtained from the polymers were collected over a range of the scattering angle between the incident beams and diffracted beam (2θ) from ≈1.8° to 30° at steps (Δ2θ) of 0.04°, each measured for 10 s. Changes in position and intensity of peaks of the diffracted X-rays were measured upon crystallization and melting of the polymers.

## Results and Discussion

### Investigating the Crystallization of the H – *T*_m_ Form of DMDS-*alt*-DVE Alternating Copolymer

Based on a combination of techniques such as a PLOM, X-ray scattering, and conventional DSC employing scan rates of 5 °C/min, the crystallization behavior of the alternating copolymer DMDS-*alt*-DVE was studied recently.^31^ The polymorphic nature of this polymer was established, and two different polymorphic forms were identified, having distinctly different melting temperatures (*T*_m1_ and *T*_m2_, respectively). The low-melting temperature polymorph (L – *T*_m_) was reported to have a *T*_m1_ of ca. 68 °C, while the high-melting polymorph (H – *T*_m_) had a *T*_m2_ of ca. 81 °C. A table reporting approximate crystallization and melting temperatures obtained by the different calorimeters and the corresponding scan rates can be found in the Supporting Information (see Table S1).

**Figure 1:**
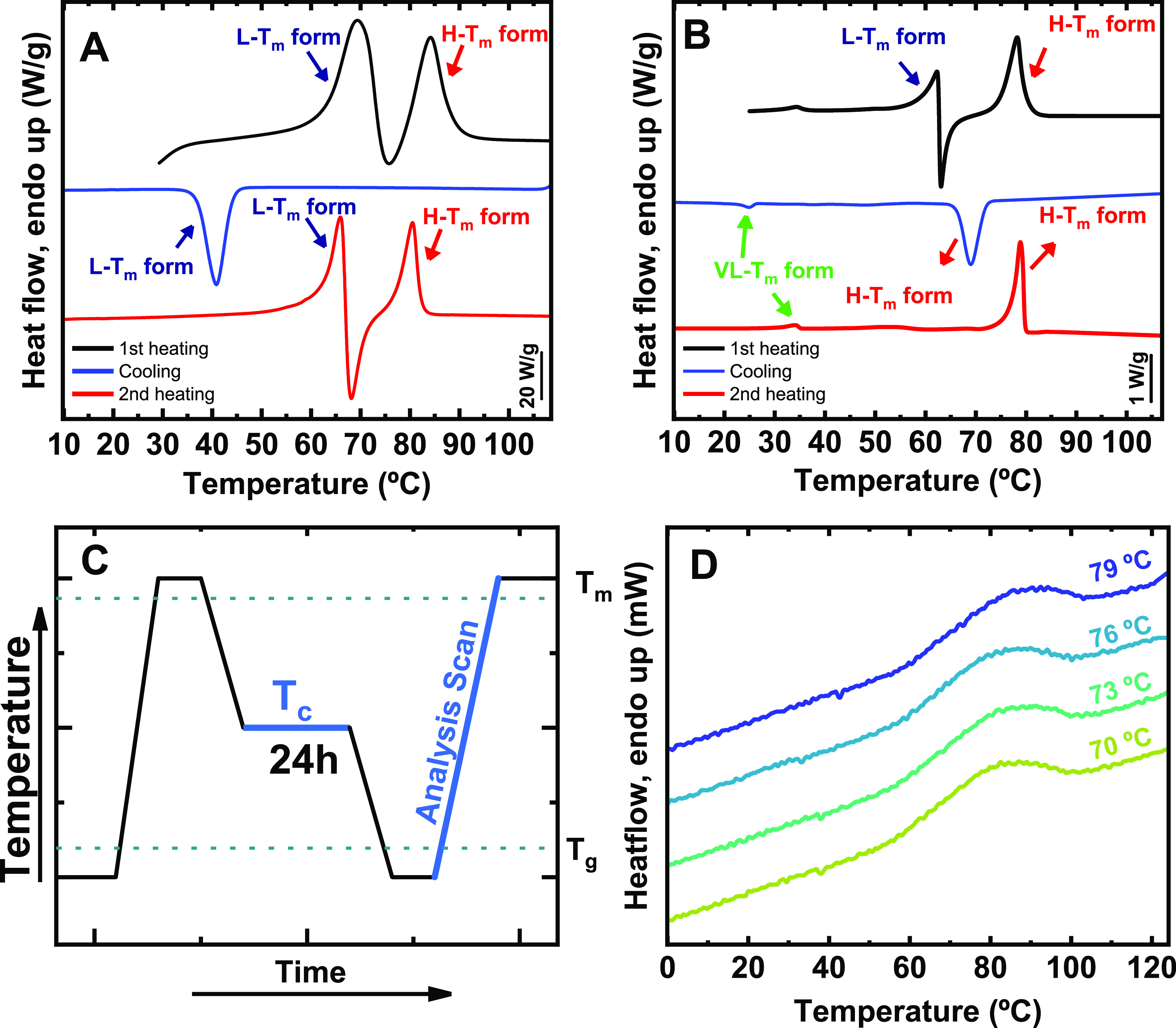
Thermal behavior of the DMDS-*alt*-DVE alternating copolymer determined via DSC, μ-DSC, and FSC. (A) DSC scans at 20 °C/min; (B) μ-DSC scans at 0.2 °C/min. (C) Thermal protocol employed for isothermal crystallization in FSC. (D) FSC heating scans (“Analysis Scan”) after isothermal crystallization for 24 h at varying *T*_c_. The arrows point to the peaks corresponding to H – *T*_m_, L – *T*_m_, or VL – *T*_m_ forms, respectively and the curves correspond to: first heating (black), cooling (blue), and second heating (red).”

In the present study, non-isothermal DSC experiments were carried out, employing heating and cooling scan rates of 20 °C/min. The resulting scans presented no detectable differences from those obtained at a slower scan rate of 5 °C/min.^31^ The corresponding DSC results of the heating and cooling runs are reported in Figure 1A. During heating, the DMDS-*alt*-DVE alternating copolymer melted at *T*_m1_ ≈ 66 °C. Following this initial melting, the sample underwent a cold-crystallization step at *T*_cc_ ≈ 68 °C, followed by a second melting at *T*_m2_ ≈ 81 °C.

According to the data reported in Figure 1A, the first endotherm (i.e., *T*_m1_) is attributed to the melting of the low-melting temperature polymorph, the L – *T*_m_ form. After the L – *T*_m_ has melted, the sample re-crystallized at *T*_cc_ (in a cold-crystallization process) into the high-melting temperature polymorph, the H – *T*_m_ form, which melted at higher temperatures (i.e., at *T*_m2_). Interestingly, when cooling from the molten state, only a single crystallization peak was observed at a crystallization temperature of *T*_c_ ≈ 40 °C, indicating that only one of the polymorphs, i.e., the L – *T*_m_ form, crystallized during cooling, as demonstrated in our previous work.^31^ Thus, by cooling from the molten state at 20 °C/min, only the L – *T*_m_ form was generated. The second heating scan in Figure 1A further supports this conclusion, showing similar behavior to that observed during the first heating. Upon heating, the L – *T*_m_ phase melted, the sample re-crystallized (cold-crystallization) and transformed into the H – *T*_m_ phase, and finally, the H – *T*_m_ phase melted. This sequential behavior occurred because the molten L – *T*_m_ phase provided a non-equilibrated melt with the memory of the previous ordered state, which assisted nucleation of the H – *T*_m_ form. That is, the formation of the H – *T*_m_ form was initiated by a kind of self-seeding^31,32^ from the memory of the molten L – *T*_m_ form. Interestingly, when using a conventional DSC, identical results were obtained for all scanning rates ranging from 1 to 50 °C/min. That is, crystallization of the H – *T*_m_ form was not achieved at any of these scanning rates when cooling the sample from the molten state at *T* > *T*_m2_.^31^ Furthermore, our findings are consistent with Ostwald’s rule of stages, which describes the sequential transformation of DMDS-*alt*-DVE as crystallization progresses. This phenomenon suggests that the first polymorph to crystallize from a polymer melt is the closest in structure to the amorphous state and differs the least in energy, eventually transforming into the more stable form.^33,34^

It is crucial to study the influence of the scanning rate on the formation of the H – *T*_m_ form to understand if its direct formation from the melt is impossible even at an extremely slow cooling rate or if the formation of the H – *T*_m_ form can also be initiated from the molten state at *T* > *T*_m2_, for example, by heterogeneous nucleation. In this work, complementary experiments have been performed at rates slower than 1 °C/min. By employing μ-DSC, we performed non-isothermal experiments at a cooling rate of 0.2 °C/min in the same manner as a conventional DSC. The sample was heated for 3 min to a temperature thirty degrees above *T*_m2_, slowly cooled to a temperature below *T*_g_, and subsequently reheated above *T*_m2_.

Figure 1B shows the results of these μ-DSC experiments. The first heating scan did not exhibit any significant differences compared to the results obtained by standard DSC for rates between 1 to 50 °C/min (see also Figure 1A). The lower observed melting peak corresponds to the L – *T*_m_ form, and the higher melting peak corresponds to the H – *T*_m_, as indicated by arrows in Figure 1B. During cooling the sample at 0.2 °C/min, a single crystallization peak was observed at a rather high temperature, with an exothermic peak at *T*_c_ ≈ 69 °C. Strikingly, this crystallization temperature is higher than *T*_m1_ measured for the L – *T*_m_ phase, indicating that this crystallization peak cannot correspond to the crystallization of the L – *T*_m_ form. Thus, it can only be due to the crystallization of the H – *T*_m_ phase. This conclusion is further supported by the absence of any melting peak associated with the L – *T*_m_ during the second μ-DSC heating scan in Figure 1B. A single melting point was observed during the second heating, which coincided with the melting temperature of the H – *T*_m_ phase, confirming that the H – *T*_m_ form crystallized at ca. 69 °C. The results of Figure 1B indicate that in the μ-DSC experiments, the H – *T*_m_ form of the alternating copolymer DMDS-*alt*-DVE can be obtained through slow cooling (i.e., at 0.2 °C/min) of the molten sample.

In light of these results, a series of experiments were carried out to explore if the H – *T*_m_ form can also be achieved by isothermal crystallization. To this end, we used FSC, a technique that allows changing temperatures so rapidly that any crystallization can be excluded during the temperature change.^35−41^ For such experiments, we used a rate of 1000 °C/s. At such a cooling rate, we did not detect any crystallization, as will be shown later in the section on the influence of the cooling rate. To erase thermal history, the sample was first heated above the maximum melting point. Next, the sample was rapidly cooled to various values of *T*_c_ above the *T*_m1_ of the L – *T*_m_ form. At *T*_c_ > *T*_m1_, only the H – *T*_m_ form can crystallize. At these various values of *T*_c_, the sample was then kept for a long time, i.e., 24 h. Subsequently, the sample was rapidly cooled to a temperature below *T*_g_ and heated again above the highest melting temperature of the material. The applied thermal protocol is presented schematically in Figure 1C.

Each of these FSC heating scans (the “Analysis Scan”) after isothermal crystallization at various values of *T*_c_ (see Figure 1D) showed a single melting endotherm at *T*_m_>80 °C, which we associated with the melting temperature *T*_m2_ of the H – *T*_m_ form. Thus, we have demonstrated that by providing sufficiently long times for crystallization, the H – *T*_m_ form of DMDS-*alt*-DVE can also be obtained via isothermal crystallization.

### Morphology of the H – *T*_m_ Form of the DMDS-*alt*-DVE Alternating Copolymer

**Figure 2:**
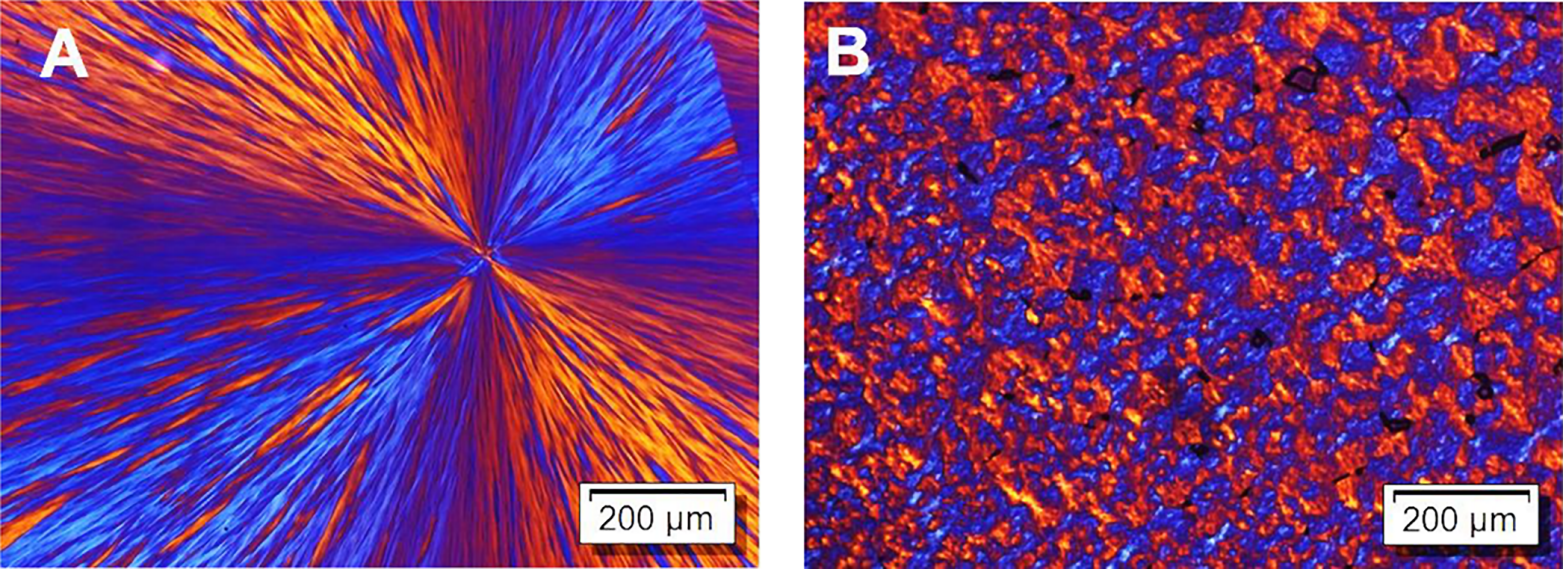
PLOM images of the H – *T*_m_ form of the alternating copolymer DMDS-*alt*-DVE after (A) isothermal crystallization at *T*_c_ = 70 °C for ca. 32 h and (B) after self-seeding from the L – *T*_m_ form and isothermal crystallization at *T*_c_ = 70 °C for 30 min.

The morphology and structure of a crystalline phase can be influenced by several factors, including nucleation and growth kinetics and the thermal protocol applied to the sample. As a result, depending on the thermal protocol used, the same crystallographic phase can reveal different morphologies. To generate the H – *T*_m_ crystalline phase directly from the melt, an experiment was carried out using PLOM on a thick film of ca. 50 μm. To erase thermal history, the sample was first heated thirty degrees above the maximum *T*_m_ and then cooled at 50 °C/min to *T*_c_ = 70 °C, where it was allowed to crystallize for ca. 32 h. Interestingly, as shown in Figure 2A, a single large spherulite was obtained within an area of ca. 1 mm^2^. Such large spherulites require an extremely low nucleation probability, i.e., very few nucleation sites were generated at *T*_c_ = 70 °C when cooling the sample from the molten state according to the procedure described above. For comparison, Figure 2B shows the H – *T*_m_ form obtained with the help of the melt memory of the previous crystalline state of the L – *T*_m_ form for an assisted generation of the H – *T*_m_ form. First, the sample was heated to erase thermal history and then cooled at 50 °C/min to *T*_c_ = 40 °C for 30 min, where the L – *T*_m_ form crystallized. Subsequently, the temperature was increased in order to melt the L – *T*_m_ form and to crystallize the H – *T*_m_ form at *T*_c_ = 70 °C for 30 min, yielding the different morphology shown in Figure 2B. In this case, a large number of very small impinged spherulites were observed. This nucleation density was comparable with the one previously reported for thin films of ca. 100 nm.^31^ Due to the melt memory of the molten L – *T*_m_ form, a large number density of self-nucleation sites was provided, resulting in a large number of small crystallites of the H – *T*_m_ form. Tentatively, we attribute the very few nucleation sites observed when cooling the sample directly from the molten state at *T* > *T*_m2_ to heterogeneous nuclei with a number proportional to the sample volume. We note that in thin films, upon cooling from the molten state at *T* > *T*_m2_, we never observed crystallization of the H – *T*_m_ form. Assuming that thin and thick films contain the same number of heterogeneous nuclei per unit volume, we expect that, compared to the previous examined thin films, the here studied thick films contain 1000 times more heterogeneous nuclei.

It should be noted that the spherulites have a positive sign as indicated by the colors generated when a lambda plate (i.e., a red-sensitive plate) is inserted at a 45° angle with respect to the polarization direction, as done in the present case. A positive spherulite has a larger refractive index (*n*_r_) in the radial direction than in the tangential direction (*n*_t_ < *n*_r_). Most polymers exhibit negative spherulites, but there are a few examples where polymers can display, depending on the crystallization conditions, positive spherulites, like isotactic polypropylene, poly(ethylene terephthalate), and poly(hydroxy butyrate).^42,43^ The exact origin of the sign of the spherulite is beyond the scope of the present work, requiring further morphological investigations.

### Forming and Characterizing the VL – *T*_m_ Form of the DMDS-*alt*-DVE Alternating Copolymer

To further investigate the influence of the cooling rate on the crystallization behavior of the alternating copolymer DMDS-*alt*-DVE, FSC experiments were conducted as this technique allows for very fast cooling rates. Noteworthy results were obtained by such non-isothermal crystallization experiments performed at various cooling rates but for a constant heating rate of 1000 °C/s. The samples were initially heated thirty degrees above the maximum *T*_m_ to erase any thermal history, then cooled to a temperature below *T*_g_, and heated again to temperatures well above *T*_m_.

**Figure 3:**
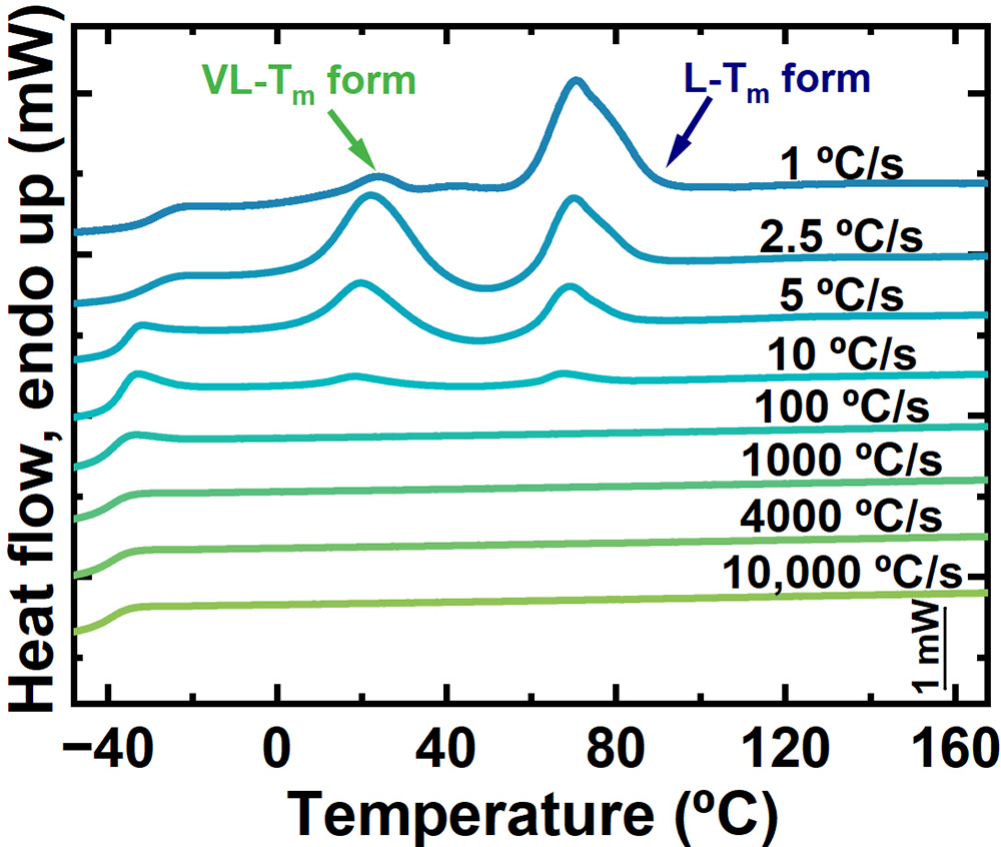
Thermal behavior of alternating copolymer DMDS-*alt*-DVE under non-isothermal conditions via FSC. The arrows indicate the distinct phases found during heating at a constant rate of 1000 °C/s, pointing to the peaks corresponding to the L – *T*_m_ and VL – *T*_m_ forms, respectively.

Figure 3 shows the FSC heating scans (obtained at a constant heating rate of 1000 °C/s) after cooling the samples at different rates as indicated next to each heating curve. For cooling rates faster than 100 °C/s, no signs of crystallization were detected during cooling, corroborated by the absence of melting peaks in the heating scans. Curves corresponding to cooling rates faster than 100 °C/s only showed a glass transition below ca. −40 °C. Thus, for such fast cooling rates, the material remained amorphous.

For cooling rates of 10, 5, and 2.5 °C/s, the heating scans of the samples showed a sequence of melting–recrystallization–melting processes. During the second heating scan, two distinct melting peaks, separated by a crystallization peak, were observed. The first melting peak was detected around 25 °C, followed by a crystallization peak around 45 °C and the second melting peak around 69 °C. The latter two peaks coincide approximately with the crystallization and melting of the L – *T*_m_ form observed with both DSC and μ-DSC, consistent with a previous study.^31^ Outstandingly, an additional polymorphic form could be observed that is distinct from the already identified H – *T*_m_ and L – *T*_m_ forms. The endotherm at *T*_m, VL_ ≈ 25 °C does not correspond to any previously reported phase, suggesting the existence of a third polymorphic form for the alternating copolymer DMDS-*alt*-DVE. When cooling at rates faster than 1 °C/s and slower than 100 °C/s, the newly discovered very low-temperature form melts and re-crystallizes into the L – *T*_m_ form, which subsequently melts. We refer to this additional phase as the very low melting temperature form (VL – *T*_m_ form or crystalline VL – *T*_m_ phase). For a cooling rate of 1 °C/s, the peak at *T*_m, VL_ ≈ 25 °C became smaller, and the one at *T*_m1_ ≈ 69 °C became more prominent. This behavior is consistent with the results shown above, for example, in Figure 1B. Microcalorimetry results indicated a tiny peak at *T*_m_ ≈ 20 °C, suggesting that a very small amount of the VL – *T*_m_ form was generated during slow cooling.

After identifying this additional polymorphic phase (VL – *T*_m_ form), experiments were conducted to generate each of the three phases individually and to explore whether they can co-exist. To generate each phase individually, the samples were initially heated well above the maximum *T*_m_ to erase any thermal history (e.g., to 170 °C).

**Figure 4:**
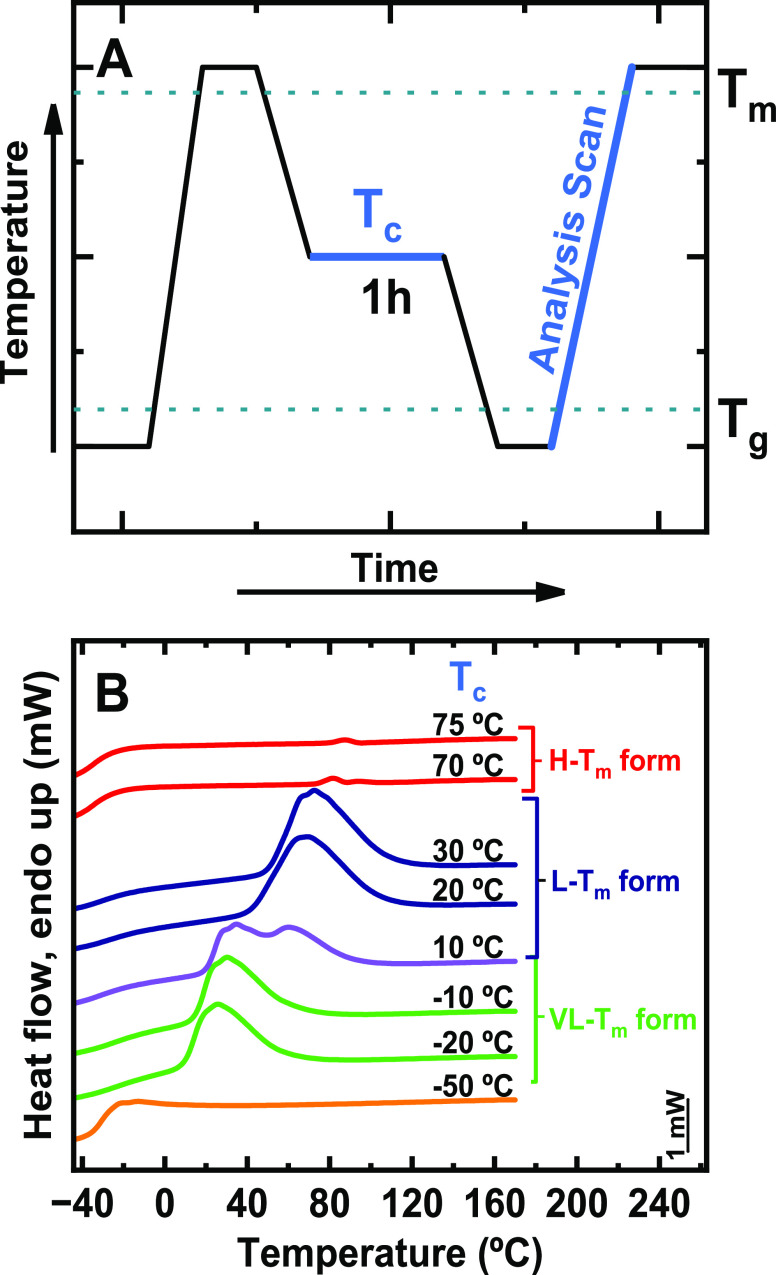
(A) Thermal protocol for isothermal crystallization experiments employed at varying *T*_c_. (B) FSC heating scans at a heating rate of 4000 °C/s, after 1 h isothermal crystallization at the indicated values of *T*_c_. The brackets point to the range of temperatures corresponding to the H – *T*_m_, L – *T*_m_, or VL – *T*_m_ forms, respectively.

To avoid crystallization during cooling, samples were then rapidly cooled to different *T*_c_ values, where they were allowed to isothermally crystallize for 1 h. Finally, the samples were rapidly cooled below *T*_g_. For each *T*_c_, the subsequent FSC heating scan, denoted as “Analysis Scan” in Figure 4A, revealed the melting of the crystals formed during the 1 h crystallization at *T*_c_. We have chosen heating and cooling rates of 4000 °C/s for the thermal protocol of Figure 4A.

Figure 4B shows the FSC heating curves measured after 1 h isothermal crystallization at the values of *T*_c_ indicated next to each curve. At *T*_c_ = −50 °C, the sample was below *T*_g_; no crystallization occurred. Upon heating at 4000 °C/s, only the glass transition was observed at approximately −37 °C. For a *T*_c_ range from −20 to −10 °C, a broad melting endotherm around *T*_m, VL_ ≈ 25 °C could be observed. For a *T*_c_ range from 20 to 30 °C, another endotherm with a higher melting point of *T*_m1_ ≈ 68 °C was found. The peak at around 25 °C corresponds to the melting of VL – *T*_m_ crystals, and that at about 68 °C corresponds to the melting of L – *T*_m_ crystals as these values align well with previously found ones. Finally, for *T*_c_ = 10 °C, we could observe the convolution of two endotherms; hence, both VL – *T*_m_ and L – *T*_m_ polymorphs crystallized at that temperature. Interestingly, at a first glance on Figure 4B, no significant endotherms could be detected after isothermal crystallization at *T*_c_ = 70 °C. However, if the curve was magnified, a weak endotherm could be observed with a melting point of around 81 °C, associated with melting of crystals of the H – *T*_m_ form.

To summarize, the individual generation of each of the phases was accomplished. Through isothermal crystallization at appropriate values of *T*_c_, samples with only one or two crystalline forms can be produced. Individual generation of the VL – *T*_m_ form and the L – *T*_m_ form can be mainly achieved by isothermal crystallization after rapidly cooling the sample (e.g., at rates faster than 100 °C/s) to a very low *T*_c_. Exclusive generation of the H – *T*_m_ form can be achieved either by slow cooling from the melt or by isothermal crystallization over long periods at *T*_c_ > *T*_m1_.

Having accomplished the generation of each phase individually by employing the protocols described above, we shift the focus to observing all three distinct forms simultaneously within the same sample. The ability of the material to adopt different crystalline structures under various thermal conditions, potentially leading to different properties, is of interest for tailoring the material properties required for specific applications. For this particular purpose, we devised a complex thermal protocol. However, it is worth noting that there are multiple alternative protocols suitable for accomplishing three distinct forms simultaneously within a sample. Nonetheless, the protocol described here is illustrative of the tunable thermal properties of this alternating copolymer.

**Figure 5:**
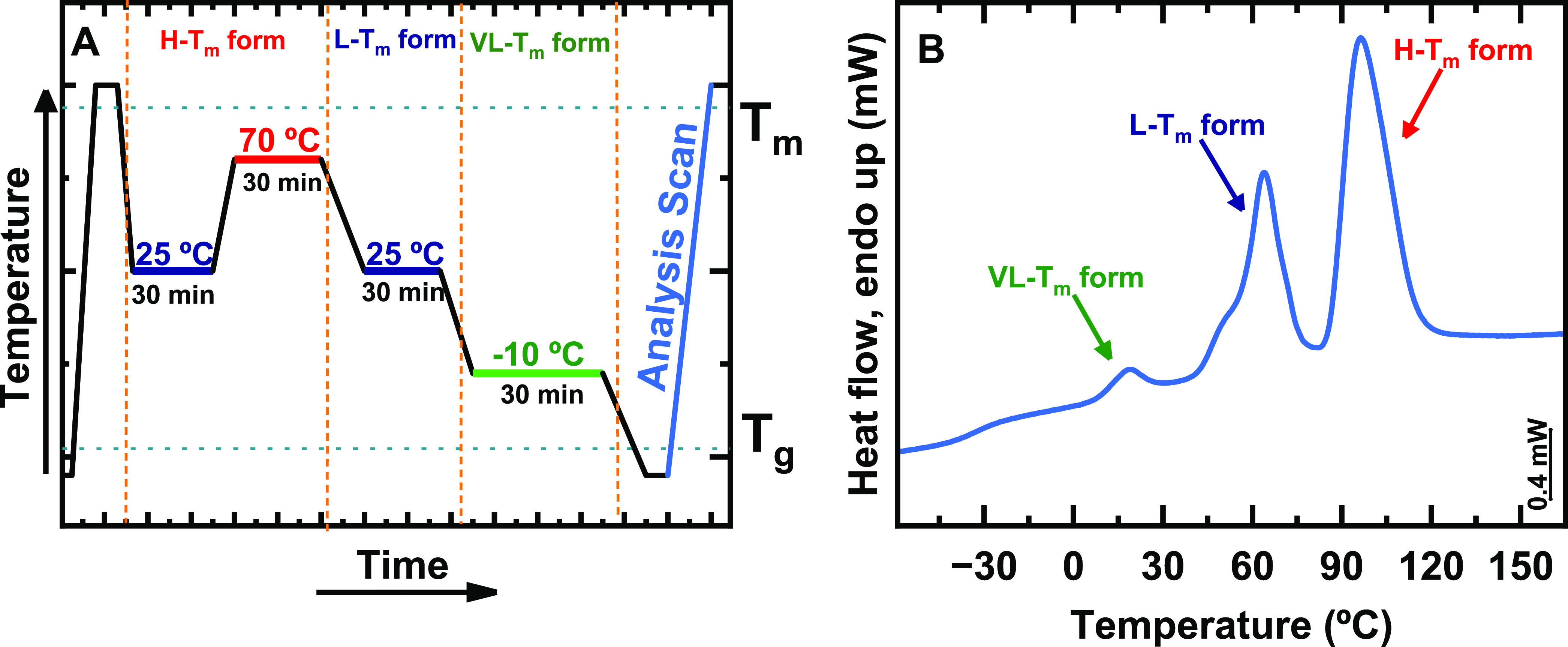
FSC experiments on alternating copolymer DMDS-*alt*-DVE. (A) Thermal protocol employed to achieve all three polymorphs of this alternating copolymer within the same sample. (B) FSC results obtained during the analysis (heating) scan shown in the protocol described in (A). All heating and cooling rates for this experiment were 1000 °C/s. The arrows point to the endothermic peaks corresponding to melting of the VL – *T*_m_, L – *T*_m_, and H – *T*_m_ forms, respectively.

In the protocol outlined in Figure 5A, depending on the desired outcome, the indicated crystallization temperatures can be adjusted. To achieve all forms within the same sample, the key requirement is that the highest melting phase (H – *T*_m_) has to be formed first. In the following, the form with an intermediate melting temperature (L – *T*_m_) and finally the one with the lowest melting temperature (VL – *T*_m_) can be generated. For the employed thermal protocol, we have chosen 1000 °C/s for all heating and cooling rates. Following the protocol of Figure 5A, the sample was first heated thirty degrees above the maximum *T*_m_ to erase thermal history, then rapidly cooled (at 1000 °C/s) to *T*_c_ = 25 °C, and kept at that temperature for 30 min. During this isothermal crystallization step, we generated the L – *T*_m_ form. The sample was then rapidly heated to *T*_c_= 70 °C where it was kept for 30 min, and part of the sample was crystallized in the H – *T*_m_ form. The L – *T*_m_ crystals formed in the previous step melted during heating, but the memory of the previous crystalline state assisted generation of the H – *T*_m_ form at *T*_c_= 70 °C. To re-generate the L – *T*_m_ form, the sample was rapidly cooled to *T*_c_ = 25 °C and kept there for 30 min. After this stage, both the H – *T*_m_ and L – *T*_m_ have been formed within the same sample. However, the sample was not yet crystallized completely, and crystallization of the VL – *T*_m_ form was achieved by rapidly cooling to *T*_c_= −10 °C and keeping the sample at this low isothermal crystallization temperature for 30 min. Finally, the sample was rapidly quenched (at 1000 °C/s) below *T*_g_. After all three forms were generated in the same sample, an FSC heating scan (denoted as “Analysis Scan”, in Figure 5A) was performed to determine the individual melting temperatures.

Figure 5B shows the corresponding FSC analysis scan performed at a rate of 1000 °C/s, where three melting peaks can be observed. The peak at *T*_m,VL_ ≈ 20 °C corresponds to the melting of the VL – *T*_m_ form, the peak at *T*_m1_ ≈ 65 °C corresponds to the melting of the L – *T*_m_ form, and finally the peak at *T*_m2_ ≈ 95 °C corresponds to the melting of the H – *T*_m_ form. We note that due to the fast heating scan, the values of the melting peaks are somewhat higher than those observed for slow heating rates. We conclude that by applying properly-tailored thermal protocols, we can generate each phase individually or produce a sample where two or all three of them co-exist.

### Crystalline Structures of the Alternating Copolymer DMDS-*alt*-DVE at Various Low Temperatures and Related Changes in Time

**Figure 6:**
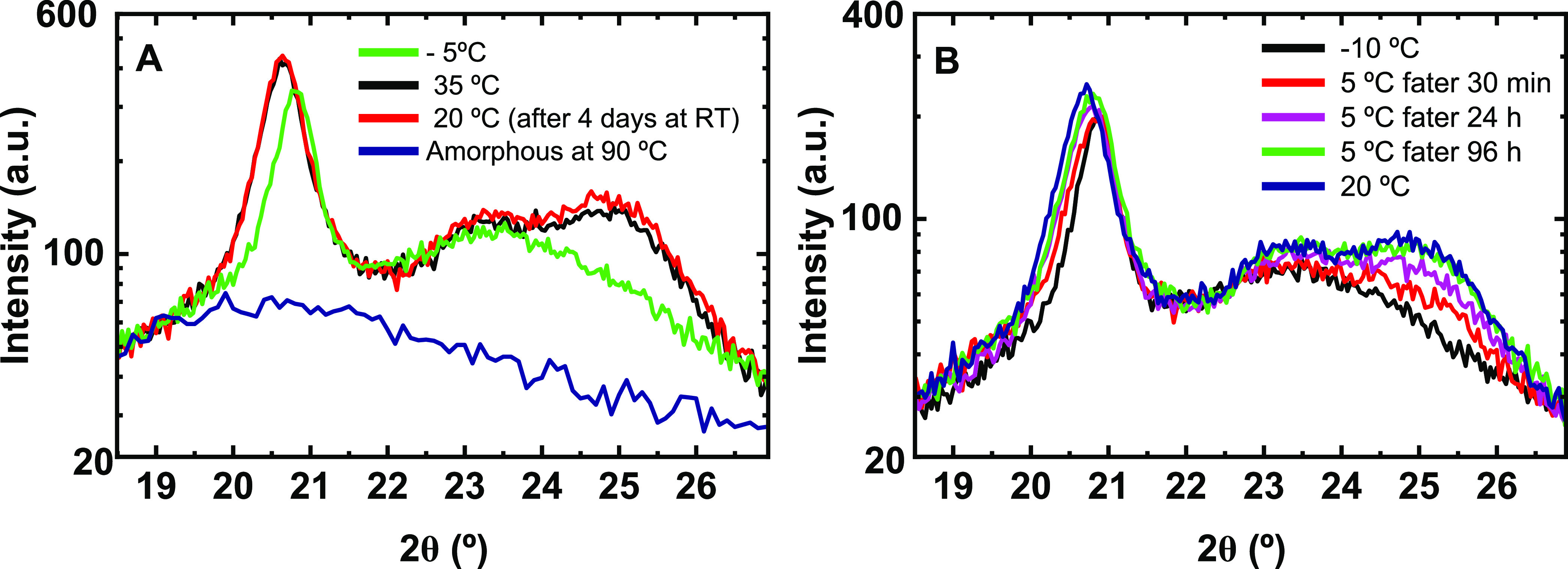
Isothermal WAXS diffractograms measured at (A) −5 °C (green curve) and 35 °C (black curve) by increasing the temperature in steps of 5 °C after being stored for four days at RT = 20 °C (red curve) and at 90 °C, (amorphous background, blue curve) and (B) −10 °C (black curve) in steps of 5 °C up to 5 °C and left for four days (after 30 min = red curve; 24 h = purple curve; 96 h = green curve) and after heating it to 20 °C (blue curve).

Our WAXS experiments aimed to explore differences in crystalline structures between the VL – *T*_m_ and L – *T*_m_ forms. The scattering patterns for the L – *T*_m_ and H – *T*_m_ forms have been reported previously.^31^ Samples were prepared as described in the experimental section. First, thermal history was erased by heating the sample to 100 °C for 1 min, which is above the maximum *T*_m2_. Subsequently, the sample was quenched by rapidly depositing it onto a brass block cooled to −20 °C, where it was kept for 5 min. According to the FSC results discussed above, the VL – *T*_m_ form was generated by this thermal treatment. To identify the anticipated crystalline structures, the sample was mounted in the diffractometer’s holder, which had been previously cooled to −10 °C. Starting at this low temperature, ten isothermal WAXS spectra were measured in steps of 5 °C up to 35 °C. Two of these spectra are shown in Figure 6A. The sample was eventually cooled back to room temperature (RT = 20 °C) and measured again after being kept at RT for 4 days. As shown in Figure 6A, the spectrum measured at 20 °C did not differ from the one measured before at 35 °C, indicating the stability of the underlying crystalline L – *T*_m_ phase. Notably, at temperatures below ca. 5 °C, the measured spectra showed clear differences from the ones observed at temperatures above ca. 5 °C. As an example, we show in Figure 6A the spectrum measured at −5 °C. In particular, the position of the dominant peak differed by about (0.2 ± 0.05)^°^, and the peak at ca. 25° was not observed at temperatures below ca. 5 °C. We conclude that the VL – *T*_m_ form generated at −20 °C has transformed into the L – *T*_m_ form at temperatures above 5 °C.

In order to explore if this transformation was a rapid or a slow process, we examined the temporal evolution of the spectrum measured at 5 °C. As can be seen in Figure 6B, the transformation of the VL – *T*_m_ form into the L – *T*_m_ form was slow and required more than ca. 30 h. Besides the progressive shifting of the dominant peak to lower scattering angles, two distinguishable peaks emerged out of the initially “broad” peak located at around 23.5°. We tentatively conclude that the VL – *T*_m_ form is probably metastable and contains less perfectly ordered crystalline structures. While the similarities of the peak positions may indicate similar unit cell parameters, the difference in the scattering intensities may result from differences in chain packing. For the VL – *T*_m_ form, which has the lowest melting temperature, we may expect a rather imperfect or less ordered structure. However, at present, all details of the crystalline structure of the three detected polymorphs are still not resolved.

## Conclusions

This work explored the different polymorphic forms generated by the alternating copolymer DMDS-*alt*-DVE at low temperatures. We demonstrated that under specific thermal protocols, this polymer has the ability to crystallize into up to three polymorphic forms. Besides the previously presented L – *T*_m_ and H – *T*_m_ forms,^31^ the alternating copolymer DMDS-*alt*-DVE possesses an additional polymorphic form that can be generated at temperatures well below room temperature (i.e., the VL – *T*_m_ polymorph). Due to clear differences in their melting temperatures and certain microstructural features, these polymorphs are easily distinguishable.

Interestingly, as shown here, the most stable crystallographic form (the H – *T*_m_ form) of DMDS-*alt*-DVE can also be established directly from the molten state. Given that there exist appropriate heterogeneous nuclei, the H – *T*_m_ form can be generated by either cooling the sample very slowly from the melt (e.g., at a rate of 0.2 °C/min or less) or by cooling the sample rapidly to a temperature above the melting temperature of the L – *T*_m_ form and keeping it there for long times (e.g., 24 h or longer). However, even without any heterogeneous nuclei, the H – *T*_m_ form can be generated rapidly by first preparing the L – *T*_m_ form and employing the melt memory of the previous crystalline state of the L – *T*_m_ form for an assisted nucleation of the H – *T*_m_ form.

Finally, based on the knowledge of the melting temperatures of the different polymorphs and the corresponding nucleation probability, including concepts of melt memory and self-nucleation, we have successfully devised an appropriate thermal protocol, which allows to generate samples that contain only one, two, or all three crystalline forms. Thus, our results demonstrate the tunability and controlled formation of different crystalline polymorphs with potentially different properties within a single polymer sample. Further studies on each polymorph generated individually may reveal differences in mechanical or optical properties, opening a promising avenue for the exploitation of the specific properties of each polymorph.

## Supporting Information Available

A brief description of the synthesis of the alternating copolymer DMDS-*alt*-DVE, a brief description on the obtention of the molecular weight distribution, and a table containing calorimetric data of DMDS-*alt*-DVE obtained for different techniques and rates (PDF)

## References

[ref1] Cramer N. B.; Scott J. P.; Bowman C. N. Photopolymerizations of Thiol-Ene Polymers without Photoinitiators. Macromolecules 2002, 35, 5361–5365. 10.1021/ma0200672.

[ref2] Hoyle C. E.; Bowman C. N. Thiol–Ene Click Chemistry. Angew. Chem., Int. Ed. 2010, 49, 1540–1573. 10.1002/anie.200903924. 20166107

[ref3] Kade M. J.; Burke D. J.; Hawker C. J. The Power of Thiol-Ene Chemistry. J. Polym. Sci., Part A: Polym. Chem. 2010, 48, 743–750. 10.1002/pola.23824.

[ref4] Quoc Le C.; Schmutz M.; Chemtob A. Ab Initio Batch Emulsion Thiol-Ene Photopolymerization. Macromolecules 2020, 53, 2369–2379. 10.1021/acs.macromol.0c00265.

[ref5] Resetco C.; Hendriks B.; Badi ab N.; Du Prez F. Thiol-Ene Chemistry for Polymer Coatings and Surface Modification-Building in Sustainability and Performance. Mater. Horiz. 2017, 4, 1041–1053. 10.1039/c7mh00488e.

[ref6] Jasinski F.; Lobry E.; Tarablsi B.; Chemtob A.; Croutxé-Barghorn C.; Le Nouen D.; Criqui A. Light-Mediated Thiol-Ene Polymerization in Miniemulsion: A Fast Route to Semicrystalline Polysul Fide Nanoparticles. ACS Macro Lett. 2014, 3, 958–962. 10.1021/mz500458s. 35596368

[ref7] Lowe A. B. Thiol–Ene “Click” Reactions and Recent Applications in Polymer and Materials Synthesis: A First Update. Polym. Chem. 2014, 5, 4820–4870. 10.1039/C4PY00339J.

[ref8] Jasinski F.; Schweitzer J.; Fischer D.; Lobry E.; Croutxe C.; Schmutz M.; Le Nouen D.; Criqui A.; Chemtob A. Thiol–Ene Linear Step-Growth Photopolymerization in Miniemulsion: Fast Rates, Redox-Responsive Particles, and Semicrystalline Films. Macromolecules 2016, 49, 1143–1153. 10.1021/acs.macromol.5b02512.

[ref9] Le C. M. Q.; Schrodj G.; Ndao I.; Bessif B.; Heck B.; Pfohl T.; Reiter G.; Elgoyhen J.; Tomovska R.; Chemtob A. Semi-Crystalline Poly(Thioether) Prepared by Visible-Light-Induced Organocatalyzed Thiol-Ene Polymerization in Emulsion. Macromol. Rapid Commun. 2022, 43, 2100740 10.1002/marc.202100740. 34890084

[ref10] Le C. M. Q.; Vidal L.; Schmutz M.; Chemtob A. Droplet Nucleation in Miniemulsion Thiol–Ene Step Photopolymerization. Polym. Chem. 2021, 12, 2084–2094. 10.1039/D1PY00139F.

[ref11] Durham O. Z.; Krishnan S.; Shipp D. A. Polymer Microspheres Prepared by Water-Borne Thiol-Ene Suspension Photopolymerization. ACS Macro Lett. 2012, 1, 1134–1137. 10.1021/MZ300358J. 35607182

[ref12] Piorkowska E.; Rutledge G. C. Handbook of Polymer Crystallization; John Wiley & Sons, 2013.

[ref13] De Rosa C.; Auriemma F. Crystals and Crystallinity in Polymers: Diffraction Analysis of Ordered and Disordered Crystals, 1st edition; De Rosa C.; Auriemma F., Eds.; Wiley, 2013.

[ref14] Kitamura M. Strategy for Control of Crystallization of Polymorphs. CrystEngComm 2009, 11, 949–964. 10.1039/B809332F.

[ref15] De Rosa C.; Auriemma F.; Malafronte A.; Scoti M. Crystal Structures and Polymorphism of Polymers: Influence of Defects and Disorder. Polym. Cryst. 2018, 1, e10015 10.1002/pcr2.10015.

[ref16] Gentili D.; Gazzano M.; Melucci M.; Jones D.; Cavallini M. Polymorphism as an Additional Functionality of Materials for Technological Applications at Surfaces and Interfaces. Chem. Soc. Rev. 2019, 48, 2502–2517. 10.1039/C8CS00283E. 30869083

[ref17] De Rosa C.; Scoti M.; Di Girolamo R.; de Ballesteros O. R.; Auriemma F.; Malafronte A. Polymorphism in Polymers: A Tool to Tailor Material’s Properties. Polym. Cryst. 2020, 3, 1–29. 10.1002/PCR2.10101.

[ref18] Brittain H. G. Polymorphism in Pharmaceutical Solids, 2nd edition; Brittain H. G., Ed.; CRC Press: Milford, 2009; vol 192.

[ref19] Liu C.; Brandenburg J. G.; Valsson O.; Kremer K.; Bereau T. Free-Energy Landscape of Polymer-Crystal Polymorphism. Soft Matter 2020, 16, 9683–9692. 10.1039/D0SM01342K. 33000842

[ref20] Keller A.; Cheng S. Z. D. The Role of Metastability in Polymer Phase Transitions. Polymer 1998, 39, 4461–4487. 10.1016/S0032-3861(97)10320-2.

[ref21] Zheng Y.; Pan P. Crystallization of Biodegradable and Biobased Polyesters: Polymorphism, Cocrystallization, and Structure-Property Relationship. Prog. Polym. Sci. 2020, 109, 101291 10.1016/J.PROGPOLYMSCI.2020.101291.

[ref22] Wu X.; Shi S.; Yu Z.; Russell T. P.; Wang D. AFM Nanomechanical Mapping and Nanothermal Analysis Reveal Enhanced Crystallization at the Surface of a Semicrystalline Polymer. Polymer 2018, 146, 188–195. 10.1016/J.POLYMER.2018.05.043.

[ref23] Marigo A.; Marega C.; Cecchin G.; Collina G.; Ferrara G. Phase Transition II → I in Isotactic Poly-1-Butene: Wide- and Small-Angle X-Ray Scattering Measurements. Eur. Polym. J. 2000, 36, 131–136. 10.1016/S0014-3057(99)00043-9.

[ref24] Azzurri F.; Flores A.; Alfonso G. C.; Baltá Calleja F. J. Polymorphism of Isotactic Poly(1-Butene) as Revealed by Microindentation Hardness. 1: Kinetics of the Transformation. Macromolecules 2002, 35, 9069–9073. 10.1021/ma021005e.

[ref25] Cavallo D.; Kanters M. J. W.; Caelers H. J. M.; Portale G.; Govaert L. E. Kinetics of the Polymorphic Transition in Isotactic Poly(1-Butene) under Uniaxial Extension. New Insights from Designed Mechanical Histories. Macromolecules 2014, 47, 3033–3040. 10.1021/ma500281f.

[ref26] Qiao Y.; Wang Q.; Men Y. Kinetics of Nucleation and Growth of Form II to I Polymorphic Transition in Polybutene-1 as Revealed by Stepwise Annealing. Macromolecules 2016, 49, 5126–5136. 10.1021/acs.macromol.6b00862.

[ref27] Xiao W.; Wu P.; Feng J.; Yao R. Influence of a Novel β-Nucleating Agent on the Structure, Morphology, and Nonisothermal Crystallization Behavior of Isotactic Polypropylene. J. Appl. Polym. Sci. 2009, 111, 1076–1085. 10.1002/APP.29139.

[ref28] Marco C.; Gómez M. A.; Ellis G.; Arribas J. M. Activity of a β-Nucleating Agent for Isotactic Polypropylene and Its Influence on Polymorphic Transitions. J. Appl. Polym. Sci. 2002, 86, 531–539. 10.1002/APP.10811.

[ref29] Garbarczyk J.; Paukszta D.; Borysiak S. Polymorphism of Isotactic Polypropylene in Presence of Additives, in Blends and in Composites. J. Macromol. Sci. Phys. B 2002, 41, 1267–1278. 10.1081/MB-120013096.

[ref30] Horváth Z.; Sajó I. E.; Stoll K.; Menyhárd A.; Varga J. The Effect of Molecular Mass on the Polymorphism and Crystalline Structure of Isotactic Polypropylene. Express Polym. Lett. 2010, 4, 101–114. 10.3144/EXPRESSPOLYMLETT.2010.15.

[ref31] Bessif B.; Heck B.; Pfohl T.; Minh C.; Le Q.; Chemtob A.; Pirela V.; Elgoyhen J.; Tomovska R.; Müller A. J.; Reiter G. Nucleation Assisted through the Memory of a Polymer Melt: A Different Polymorph Emerging from the Melt of Another One. Macromolecules 2023, 56, 1461–1470. 10.1021/acs.macromol.2c02252.

[ref32] Sangroniz L.; Cavallo D.; Müller A. J. Self-Nucleation Effects on Polymer Crystallization. Macromolecules 2020, 53, 4581–4604. 10.1021/ACS.MACROMOL.0C00223.

[ref33] Hedges L. O.; Whitelam S. Limit of Validity of Ostwalds Rule of Stages in a Statistical Mechanical Model of Crystallization. J. Chem. Phys. 2011, 135, 164902 10.1063/1.3655358. 22047264

[ref34] Tavassoli Z.; Sear R. P. Homogeneous Nucleation near a Second Phase Transition and Ostwald’s Step Rule. J. Chem. Phys. 2002, 116, 5066–5072. 10.1063/1.1452108.

[ref35] Schick C.; Mathot V. Fast Scanning Calorimetry; Schick C.; Mathot V., Eds.; Springer Cham, 2016.

[ref36] Schick C.; Androsch R. New Insights into Polymer Crystallization by Fast Scanning Chip Calorimetry. In Fast Scanning Calorimetry; 2016; vol 91, pp 463–535.

[ref37] Schawe J. E. K.; Ag M.; Schwerzenbach C. Flash DSC 1: A Novel Fast Differential Scanning Calorimeter. 27th World Congress of the Polymer Processing Society 2011, 27, 1–4.

[ref38] Toda A.; Androsch R.; Schick C. Insights into Polymer Crystallization and Melting from Fast Scanning Chip Calorimetry. Polymer 2016, 91, 239–263. 10.1016/J.POLYMER.2016.03.038.

[ref39] Sangroniz L.; Ocando C.; Cavallo D.; Müller A. J. Melt Memory Effects in Poly(Butylene Succinate) Studied by Differential Fast Scanning Calorimetry. Polymers 2020, 12, 2796 10.3390/polym12122796. 33256010 PMC7761523

[ref40] Schawe J. E. K. Influence of Processing Conditions on Polymer Crystallization Measured by Fast Scanning DSC. J. Therm. Anal. Calorim. 2014, 116, 1165–1173. 10.1007/s10973-013-3563-8.

[ref41] Furushima Y.; Schick C.; Toda A. Crystallization, Recrystallization, and Melting of Polymer Crystals on Heating and Cooling Examined with Fast Scanning Calorimetry. Polym. Cryst. 2018, 1, e10005 10.1002/PCR2.10005.

[ref42] Caputo M. R.; Tang X.; Westlie A. H.; Sardon H.; Chen E. Y. X.; Müller A. J. Effect of Chain Stereoconfiguration on Poly(3-Hydroxybutyrate) Crystallization Kinetics. Biomacromolecules 2022, 23, 3847–3859. 10.1021/acs.biomac.2c00682. 35929661 PMC9472230

[ref43] Gedde U. W.; Hedenqvist M. S. Fundamental Polymer Science, 2nd edition; Springer Nature: Cham, Switzerland, 2019.

