# Supplementary material for: Unraveling the Complex Polymorphic Crystallization Behavior of the Alternating Copolymer DMDS-alt-DVE
Source: ACS Appl Polym Mater. 2023 Jun 9;5(7):5260–9. doi: 10.1021/acsapm.3c00684 (PMC10353521; doi:10.1021/acsapm.3c00684)
Supplement: Supplementary file 1 — ap3c00684_si_001.pdf [file ap3c00684_si_001.pdf]

Supporting Information

for

Unraveling the complex polymorphic crystallization behavior of the alternating copolymer DMDS-alt-DVE

Valentina Pirela[§], Justine Elgoyhen[§], Radmila Tomovska[§,‡], Jaime Martín[§,‡,‖], Cuong Minh Quoc Le[#], Abraham Chemtob[#], Brahim Bessif[†], Barbara Heck[†], Günter Reiter[†], Alejandro J. Müller[§,‡]*

[§] POLYMAT and Department of Polymers and Advanced Materials: Physics, Chemistry, and Technology, Faculty of Chemistry, University of the Basque Country UPV/EHU, Paseo Manuel de Lardizabal 3, 20018, Donostia-San Sebastián, (Spain).

[#]Institut de Sciences des Matériaux de Mulhouse (IS2M), UMR CNRS 7361, Université de Haute-Alsace, 15 rue Jean Starcky, Mulhouse, 68057, Cedex, (France).

[†]Institute of Physics, University of Freiburg, Hermann-Herder-Str. 3, 79104, Freiburg (Germany).

[‡] IKERBASQUE, Basque Foundation for Science, Plaza Euskadi 5, 48009, Bilbao (Spain).

[‖] Universidade da Coruña, Campus Industrial de Ferrol, CITENI, Esteiro, 15403, Ferrol, (Spain).

*Corresponding author:

*Alejandro J. Müller: alejandrojesus.muller@ehu.es

Synthesis of DMDS-*alt*-DVE

A mixture of 2,2'-dimercaptodiethyl sulfide (DMDS, 0.500 g, 3.2 mmol, 1 equiv.) and the diene monomer di(ethylene glycol) divinyl ether (DVE, 0.516 g, 3.2 mmol) was added to a 20 mL soda-lime glass vial; with respect to the monomers, an aqueous phase containing a photo-catalyst (eosin disodium, 2 mg, 0.0026 mmol, 0.02% w/w monomer), a surfactant (sodium dodecyl sulfate (SDS), 35 mg, 3.5 wt%, 13.5 mM in water) and 9 mL of phosphate buffer 10 mM (pH = 8) was then added to the monomer mixture.

To create an emulsion with an organic phase content of 10% w/w, a homogenizer (Ultra-Turrax, T25, IKA-Werke) was used to homogenize the mixture at 15,000 rpm for 10 minutes. Photo-polymerization was initiated immediately after emulsification, the reaction was conducted at room temperature in a circular photochemical reactor with a green LED strip (530 nm, 3.0 $mW \cdot cm^{-2}$). The vial was continually stirred and irradiated using a magnetic stirrer (1100 rpm) for 60 minutes.

Molecular weight distribution of DMDS-alt-DVE

The molecular weight distribution of purified DMDS-alt-DVE was obtained by GPC in N,N-dimethylformamide (DMF). The sample was prepared at 1.0 mg/mL and injected into a GPC (Agilent 1260 Infinity series with a set of three columns Polymer Laboratories ResiPore). A flow of eluent containing 10 mM lithium bromide was pumped at 0.9 mL/min through the columns controlled at 50 ºC. The system was calibrated using a set of EasiVial poly(methyl methacrylate) narrow standards. The Mw was calculated using Aligent SEC software.

DMDS-*alt*-DVE melting temperatures

The table below represents the approximate melting values for each polymorph according to the technique used. It is important to understand that these are not unique and precise values, as the scanning rates and the thermal protocol employed influence the melting point of each polymorph. This table is intended to illustrate orientative values.

Table S1. Melting and crystallization temperatures of DMDS-alt-DVE for each technique.

T (ºC) / Technique	T_m $(L - T_m)$	T_m $(H - T_m)$	T_m $(VL - T_m)$	T_{cc}	T_c
**DSC (5 ºC/min)**	≈ 68[b]	≈ 81[b]	-	≈ 69[b]	≈ 40[c]
**µ-DSC (0.2 ºC/min)**	≈ 60[a]	≈ 80[b]	≈ 35[b]	≈ 65[a]	≈ 69[c]
**DSC (50 ºC/min)**	≈ 66[b]	≈ 80[b]	-	≈ 68[b]	≈ 41[c]
**FSC (1000 ºC/s)**	≈ 68[d]	≈ 83[d]	≈ 21[d]	≈ 45[a]	-

[a] Data obtained from the first heating scan of non-isothermal experiments.
[b] Data obtained from the 2nd heating scan ("Analysis Scan") of non-isothermal experiments.
[c] Data obtained from the cooling scan of non-isothermal experiments.
[d] Data obtained from the 2nd heating scan ("Analysis Scan") of isothermal experiments.
